# Combining transcriptomics with network pharmacology to explore the mechanism of Yiqi Huoxue decoction against liver fibrosis

**DOI:** 10.1371/journal.pone.0337061

**Published:** 2025-11-26

**Authors:** Yao-Yao Mao, Ke Zhang, Dan-Dan Zhao, Jia-Wei Cui, Zhan-Dong Lin, Cong-Yue Zhang, Yue-Min Nan

**Affiliations:** 1 Department of Traditional and Western Medical Hepatology, Third Hospital of Hebei Medical University, Shijiazhuang, Hebei, People's Republic of China; 2 Hebei Provincial Key Laboratory of Liver Fibrosis in Chronic Liver Diseases, Shijiazhuang, Hebei, People's Republic of China; 3 Department of Ultrasound, The First Affiliated Hospital of Zhengzhou University, Zhengzhou, Henan, People's Republic of China; Hong Kong Baptist University, HONG KONG

## Abstract

**Background:**

Clinical practice commonly uses the Yi-qi Huo-xue formula (YQHX), a traditional Chinese herbal medicine comprising eight herbal components, to treat liver fibrosis resulting from various etiologies. Nevertheless, this formula’s specific active constituents and underlying mechanisms of action remain to be fully elucidated.

**Methods:**

The drug components of YQHX and potential targets for liver fibrosis were identified via the screening of the various databases. Qualitative and quantitative identification of chemical components of drug-containing serum by Ultra Performance Liquid Chromatography (UPLC).Liver fibrosis was induced in mice through the intraperitoneal injection of carbon tetrachloride, followed by oral administration of YQHX. RNA-Seq quantified transcriptomic profiles in liver tissue.The degree of liver fibrosis was assessed via histopathology staining, the transcription and expression of relevant proteins were analyzed. Primary cells were isolated for in vitro experiments to validate the influence of YQHX on the associated signaling pathways.

**Results:**

Network pharmacology identified IL-1β, IL-6, and TNF-α as potential targets for YQHX in treating liver fibrosis.The UPLC detected multiple potential active components. In vivo experiments showed that YQHX reduced serum AST and ALT levels in liver fibrosis-induced mice, decreased liverIL-1β, IL-6, and TNF-α levels, and improved liver fibrosis.The results of transcriptomics suggest that YQHX can reduce the expression of “collagen-activated signaling pathway,” “MyD88-dependent toll-like receptor signaling pathway,” “fibrinolysis” and “toll-like receptor 4 signaling pathway”. Furthermore, YQHX reduced the aggregation of M1 macrophages in the portal area and the deposition of α-SMA. Primary bone marrow-derived cells successfully transformed into M1 macrophages after induction, and YQHX reduced the levels of IL-1β, IL-6, and TNF-α in the supernatant of M1 macrophage culture and decreased the activation of primary hepatic stellate cells indirectly co-cultured with the supernatant. Interestingly, TLR4 agonists weakened this inhibitory effect. Both in vitro and in vivo experiments demonstrated that YQHX could inhibit the expression of the TLR4/TRAF6/MyD88 pathway in M1 macrophages.

**Conclusion:**

We reveal here the molecular mechanism and signaling pathway of YQHX in treating liver fibrosis by utilizing network pharmacology in conjunction with in vivo and in vitro experiments. The findings offer insights that may advance the clinical application of YQHX.

## Background

Liver fibrosis is an important contributor to global mortality rates, with hepatotropic virus infections such as hepatitis B and hepatitis C [1,2], metabolic-related fatty liver hepatitis, alcoholic fatty liver hepatitis, and autoimmune hepatitis being the primary causes [3]. Liver fibrosis is a common outcome of chronic liver damage, which is characterized by the accumulation of extracellular matrix (ECM) or scar tissue in the affected area [4]. The composition of liver scars remains consistent regardless of the underlying cause of injury. The development of liver fibrosis is a gradual process that requires prolonged exposure to chronic damage over several years, and the exact point at which it becomes irreversible remains uncertain. Nevertheless, emerging evidence suggests that early cirrhosis may be reversible [5]. Despite the importance of addressing the root cause, effective treatments for liver fibrosis are also currently limited [6,7].

Utilizing network pharmacology, we aimed to identify the therapeutic targets of YQHX in the context of liver fibrosis. Initial experimental findings indicate a potential association with the secretion of inflammatory factors by M1 macrophages.Recent research findings on the progression of liver fibrosis indicate that the damage-associated molecular patterns (DAMPs) generated from liver cell necrosis interact with the pattern recognition receptors (PRRs) located on the surface of macrophages. This will facilitate the conversion of macrophages into the M1 macrophage phenotype and the subsequent release of inflammatory mediators, which play a crucial role in the development of liver fibrosis [8–10]. Hepatic stellate cells (HSCs) in the Disse gap of the liver of the liver are activated by these inflammatory signals to become activated myofibroblasts, which secrete extracellular matrix proteins and produce fibrous scars [4]. Various investigations have demonstrated the potential of traditional Chinese medicine (TCM) in ameliorating liver fibrosis by either impeding the activation of the inflammatory pathways or reducing the inflammatory responses. This highlights its potential therapeutic prospects in the management of liver fibrosis [11–15,16].

YQHX is composed of eight TCMs, namely Danshen (*Radix Salviae.*), Huangqi (*Hedysarum Multijugum Maxim.*), Shanyao (*Rhizoma Dioscoreae.*), Fuling *(Poria.*), Yujin (*Curcumae Radix.*), Doukou (*Alpinia Katsumadai Hayat.*), Chishao (*Radix Paeoniae Rubra.*), and Jineijin (*Galli Gigeriae Endothelium Corneum.*). Our previous clinical study suggests that YQHX can help patients with chronic hepatitis B receiving nucleoside analog antiviral therapy achieve better reversal of liver fibrosis [17], and potential mechanisms of action were studied from different perspectives in animal experiments [18,19]. However, the precise mechanism underlying this effect remains relatively unexplored. Therefore, this research aimed to identify the targets of YQHX using network pharmacology and investigate whether YQHX exerts anti-fibrotic effects by modulating the release of inflammatory factors or the function of M1 macrophages. This hypothesis will be further assessed using a combination of in vivo and in vitro experiments.

## Methods

### Drug-Target-Disease Network construction

The active ingredients and corresponding targets of the eight TCMs that comprise YQHX were collected from the TCMSP (https://www.tcmsp-e.com) and BATMAN-TCM databases (http://bionet.ncpsb.org.cn/batman-tcm/index.php). Liver fibrosis disease targets were collected from the DisGeNET (https://www.disgenet.org/) and GeneCards databases (https://www.genecards.org/). The protein crystal structure used for molecular docking was obtained from the PDB database (https://www.rcsb.org/). Molecular docking was performed by employing the AutoDock Vina 1.2.3 software.

### Ultra-performance liquid chromatography

For orally administered traditional Chinese medicine (TCM) formulas, the components generally enter the bloodstream or are transported to the intestine. The components of the TCM that enter the blood will circulate through the bloodstream and reach the target tissues to exert their effects. Therefore, the identification of blood components (serum pharmacochemistry) can provide direct information on the active components intended to exert therapeutic effects. To further determine whether the potentially active ingredients, identified through network pharmacology, can be absorbed into the bloodstream, we performed UPLC analysis on the YQHX-containing serum. The parameters and detailed data are presented in section S1 Text.

### Animals and treatment

C57BL/6 mice (male, 6 weeks old) and Sprague-Dawley rats (male, 8 weeks old) were purchased from Beijing Huafukang Biotechnology Co., Ltd. The housing environment was controlled at 25 ± 2°C, humidity 50 ± 5%, 12-h light-dark cycle, and ad libitum access to food and water. The animal study underwent ethical review by the Ethics Center of Hebei Provincial Third Hospital (Ethics approval number: 2024−30). The mice were randomly assigned to four groups, each consisting of 10 individuals, designated as the control Group, Model Group, Single-dose Group, and Double-dose Group. Liver fibrosis was induced by the intraperitoneal injection of 10% CCl4 (CCl4: olive oil = 1:9, 0.6 mL/100 g, biw, over 8 weeks) [20]. The Model Group, Single-dose Group, and Double-dose Group received intraperitoneal injections of 10% CCl4 to induce the disease model, while the control Group received an equivalent dose of olive oil as a negative control. YQHX was administered via gavage in the 5th week of the disease model. The equivalent dose of gastric gavage based on body surface area conversion between humans and experimental animals [21].The Single-dose Group and Double-dose Group received YQHX at doses of 1.4 g and 2.8 g per 100 g of body weight, respectively, once daily. Concurrently, the Model Group received an equivalent volume of distilled water via gavage. After 8 weeks, the mice were euthanized to collect serum and liver tissue samples. Sprague-Dawley rats (male, 8 weeks old) were used exclusively for the preparation of YQHX-containing serum for cell experiments. No separate in vivo data were obtained from these animals.

### Detection of mRNA expression in liver tissue by RNA-sep

Total RNA was extracted from liver tissue, and the RNA integrity was assessed using the RNA Nano 6000 Assay Kit of the Bioanalyzer 2100 system (Agilent Technologies, CA, USA).. After the library passed quality control, the raw data was filtered to generate clean data. featureCounts 1.5.0-p3 was used to calculate the read counts mapped to each gene and to compute the FPKM for each gene. Differential expression analysis between the two comparison groups was performed using DESeq2 1.20.0. GO enrichment analysis of differentially expressed genes was conducted using clusterProfiler 3.8.1. GO terms with a corrected P value of less than 0.05 were considered significantly enriched by the differentially expressed genes.

### Extraction and culture of primary cells

Primary HSCs and primary macrophages from mice were extracted and cultured for in vitro experimentation [22,23]. The bone marrow cells were incubated in MCSF culture medium(Cat:351871,MCE,US)to induce the maturation of macrophages. The mature macrophages were divided into M0 Group, M1 Group, Blank-control Serum Group, YQHX-containing Serum Group, and YQHX-containing Serum+Neoseptin3 Group. The M1 Group, Blank-control Serum Group, YQHX-containing Serum Group, and YQHX-containing Serum+Neoseptin3 Group were induced with LPS (Cat:HY-D1056,MCE,US) to become M1 macrophages, while the M0 Group was left untreated. Flow cytometry was used to analyze the proportions. The Blank-control Serum Group, YQHX-containing Serum Group, and YQHX-containing Serum+Neoseptin3 Group were then respectively replaced with 10% blank control serum, 10% YQHX-containing serum, and 10% YQHX-containing serum + Neoseptin3 (a TLR4 agonist,Cat:231365,MCE,US) after induction. Co-culture the supernatant of each group of macrophages with HSCs indirectly. All primary cells are cultured in a 5% CO2, 37°C, 95% humidity cell culture incubator.

### Histopathology and immunofluorescence

Liver tissue was fixed with 4% paraformaldehyde and embedded using a paraffin embedding machine. Slices were then stained accordingly. For the steps involved in the hematoxylin & eosin (H&E), Masson, or Sirius Red staining, refer to the instructions of the corresponding kits. For immunofluorescence analysis of liver tissue, paraffin sections were used, and antigen retrieval was performed after hydration. Then, primary antibodies for iNOS (ab283655, Abcam, UK) and αSMA (67735–1-lg, Proteintech, China) were used for overnight incubation. After labeling with the corresponding secondary antibodies, confocal microscopy was used for observation and photography. For cell immunofluorescence analysis, cell slides were used, β-tubulin (ab231082, Abcam) was used to stain the cytoskeleton, and TLR4 (19811–1-AP, Proteintech, China) and αSMA were used to stain the macrophages and HSCs, respectively.

### Serum biochemical analysis and ELISA

The serum was obtained by centrifuging the collected whole blood samples from mice, and the levels of AST, ALT, ALP, and TBIL were measured using an automated biochemical analyzer (Chemray 240, Rayto, China). The concentrations of IL-1β, IL-6, and TNF-α in both the mouse liver tissue and cell supernatant were determined via ELISA according to the manufacturer’s instructions using a standard commercial kit (ZCIBIO, China). The absorbance readings were measured using an enzyme plate reader (SpectraMax 190, Molecular Devices, US).

### RT-PCR

RNA was isolated using the TRIzol method and subsequently converted into complementary DNA (cDNA). The housekeeping gene GAPDH was used as an internal control, and the reverse primer sequence of the target genes is provided in **Table 1**.

**Table 1:** Sequences of forward and reverse primers.

Genes	Sequences
IL-1β	F:GAAATGCCACCTTTTGACAGTG
	R:TGGATGCTCTCATCAGGACAG
IL-6	F:TAGTCCTTCCTACCCCAATTTCC
	R:TTGGTCCTTAGCCACTCCTTC
TNF-α	F:CCCTCACACTCAGATCATCTTCT
	R:GCTACGACGTGGGCTACAG
α-SMA	F:GTCCCAGACATCAGGGAGTAA
	R:TCGGATACTTCAGCGTCAGGA
TLR4	F:ACTTGATCTACCAAGCCTTGAGT
	R:GCTGGTTGTCCCAAAATCACTTT

### Western blot analysis

Proteins were extracted from tissues or cells using RIPA buffer (R0010, Solarbio, China) supplemented with protease inhibitors, and the concentration of the extracted proteins was quantified utilizing a BCA assay kit (AR0146, Boster, China). Subsequently, the proteins were separated by 10% sodium dodecyl sulfate-polyacrylamide gel electrophoresis (SDS-PAGE) and transferred onto polyvinylidene fluoride (PVDF) membranes (Millipore, MA, USA). The membranes were blocked with 5% milk powder and probed with specific primary antibodies, including αSMA (BM3902, Boster, China), COL-1 (AF7001, Affinity, Australia), TRL4 (19811–1-AP, Proteintech), MyD88 (67969–1-lg, Proteintech), TRAF6 (CY5175, Abways, China), and the internal reference GAPDH (AB0037, Abways), and incubated overnight at 4°C. Subsequently, secondary antibodies DyLight 800 (Goat Anti-Mouse IgG A23910, Goat Anti-Rabbit IgG A23920, Abbkine, China) were applied and incubated at room temperature for 2 h. Visualization of protein bands was performed using the LI-COR imaging analysis system (LICOR, US).

### Flow cytometry

To evaluate the purity of the cultured primary macrophages and induced M1 macrophages, flow cytometry was employed to label specific markers. F4/80 (ThermoFisher, US, catalog number 15-4801-82) was used to identify mature macrophages, while iNOS (Abcam, catalog number ab283655) in conjunction with F4/80 was used for the dual staining of M1 macrophages. The secondary antibody used to label iNOS was sheep anti-rabbit Alexa Fluor 488 (Abcam, catalog number ab150077). Single-cell suspensions of primary cells from the culture dish were prepared, enumerated, incubated with antibodies, washed, resuspended, filtered, and subsequently analyzed using flow cytometry.

### Statistical analysis

The data are presented as the mean ± standard deviation, and statistical analysis and graphical representation were performed using GraphPad Prime 8.0 software. The comparison between groups was conducted using the *t*-test, while the significance of the differences between the means of the samples was assessed using one-way analysis of variance (ANOVA). A p-value < 0.05 was considered statistically significant.

## Results

### Network pharmacology analysis of YQHX

**Fig 1:**
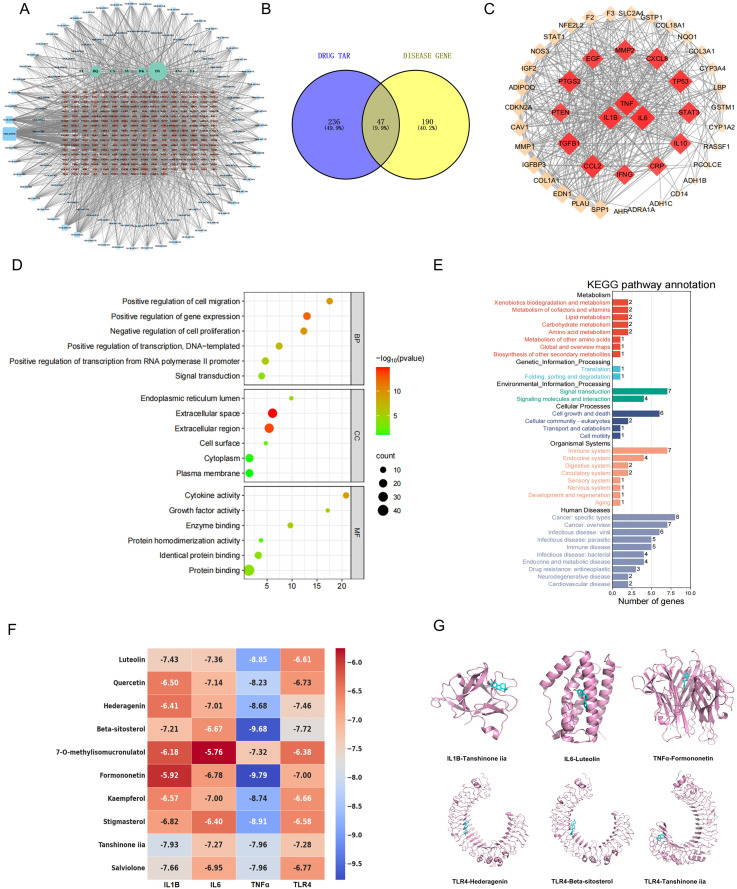
Network pharmacology analysis of potential targets and mechanisms of YQHX in alleviating liver fibrosis. (A) Active ingredients and potential therapeutic targets of YQHX. (B) Venny diagram of overlapping genes from YQHX and liver fibrosis. (C) PPI network construction. (D) GO functional enrichment analysis. (E) KEGG pathway enrichment analysis. (F) Heat map of reaction binding affinity, the lower the docking score value, the stronger the binding strength. (G) Binding mode diagram, pink represents protein, cyan represents active ingredient.

A total of 237 targets associated with liver fibrosis were examined via database screening, which revealed 108 active components from eight Chinese medicinal sources that corresponded with 283 disease targets. Among these, 47 overlapping genes were identified in the Venny map and were considered potential targets for YQHX in liver fibrosis treatment (**Fig 1A**). The Drug-Target-Disease Network illustrates the multi-component and multi-target nature of this Chinese medicine (**Fig 1B**). Analysis using the PPI dataset highlighted the involvement of IL-1β, IL-6, and TNF-α in network enrichment (**Fig 1C**). Enrichment analysis indicated that genes were primarily enriched in the GO Biological Process for “positive regulation of gene expression,” in the Cellular Component for the “extracellular space and region,” and in the Molecular Function for “protein binding” (**Fig 1D**). Furthermore, KEGG pathway analysis revealed associations with the “immune system,” “signal transduction,” “cell growth and death,” and “specific types of cancer” (**Fig 1E**).Molecular docking simulation technology is a convenient and effective tool for investigating the interaction between compounds and disease-target proteins. In this study, Vina 1.2.3 software was utilized to perform docking analyses on compounds and key proteins. The investigation focused on the 10 active ingredients that exhibited the highest correlation with the disease targets as identified through network pharmacology. Specifically, molecular docking experiments were conducted with IL-1β, IL-6, TNF-α, and its upstream receptor, TLR4. The docking scores obtained serve as indicators of binding affinity, with lower values suggesting stronger binding. Analysis of the resulting heat map revealed that the majority of binding energies exceeded −6.0 kcal/mol, which indicates favorable binding effects of these molecules to the target proteins (**Fig 1F**). The results of the molecular docking simulations indicated that Formononetin exhibited the best binding effect with TNF-α, Luteolin with IL-6, and tanshinone iia with IL-1β. Moreover, Tanshinone IIA, Hederagenin, and Beta-sitosterol emerged as the top three active ingredients capable of binding to TLR4 (**Fig 1G**).

### UPLC validation of potential active ingredients in drug-containing serum

Network pharmacology and molecular docking experiments have identified the therapeutic targets of YQHX in treating liver fibrosis and five potential active components that interact with these targets. Subsequently, we detected four components in the drug serum using UPLC, namely Luteolin, Hederagenin, Tanshinone IIA, and Formononetin. Detailed parameter information can be found in S2 Table, and the total ion chromatogram (TIC) is attached in S2 and S4 Fig. The significant potential of these compounds in the treatment of liver diseases has been widely recognized, as detailed in S3 Table.

### YQHX reduces liver inflammation and fibrosis in the CCl4-induced mouse disease model

**Fig 2:**
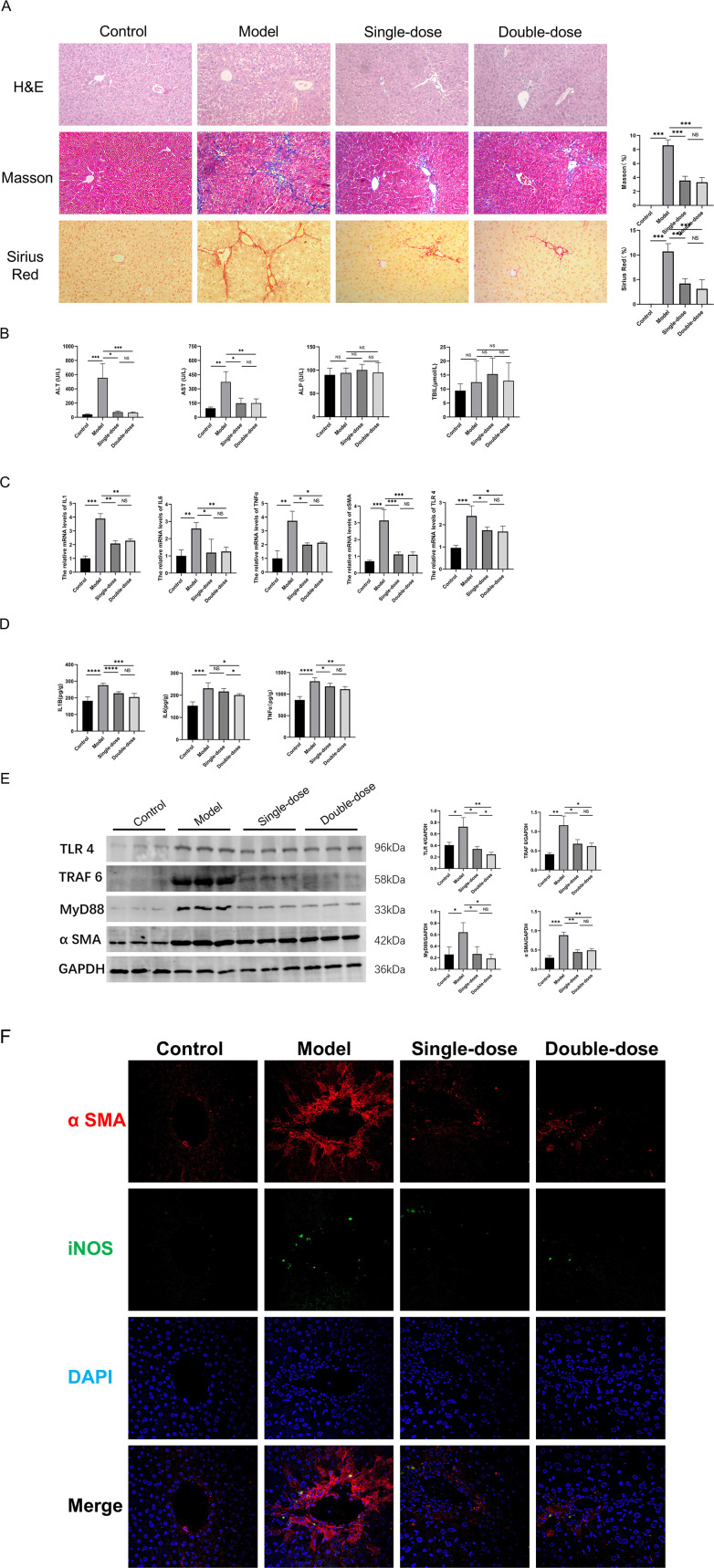
Continued.

The H&E, Masson, and Sirius Red staining experiments revealed alterations in the liver tissue morphology in Model Group mice compared to that of control Group mice. This was characterized by hepatic steatosis, cell swelling, hepatocyte lysis, necrosis, inflammatory cell infiltration, disrupted liver cord arrangement, pseudo-lobule formation, and pronounced liver fibrosis. The Single-dose Group mice exhibited reduced fat vacuoles and inflammatory cell infiltration, decreased collagen fiber deposition, and improved liver fibrosis compared to the Model Group mice. No significant difference was observed between the Double- and Single-dose Group mice. The administration of YQHX was found to decrease inflammatory cell infiltration, collagen fiber production, pseudo-lobule and fibrous septa formation, and aid in restoring the normal liver tissue structure (**Fig 2A**). Furthermore, YQHX significantly lowered the serum AST and ALT levels in mice with liver fibrosis (**Fig 2B**). PCR and ELISA analyses indicated that YQHX reduced the transcription and translation levels of IL-1β, IL-6, and TNF-α in the liver tissue of the mouse disease models (**Fig 2C**, **2D**). PCR and western blot results revealed that YQHX notably decreased the expression of α-SMA in the liver tissue at the transcription and translation levels (**Fig 2C**, 2E).

### YQHX regulates several pathways related to the occurrence and development of liver fibrosis

**Fig 3:**
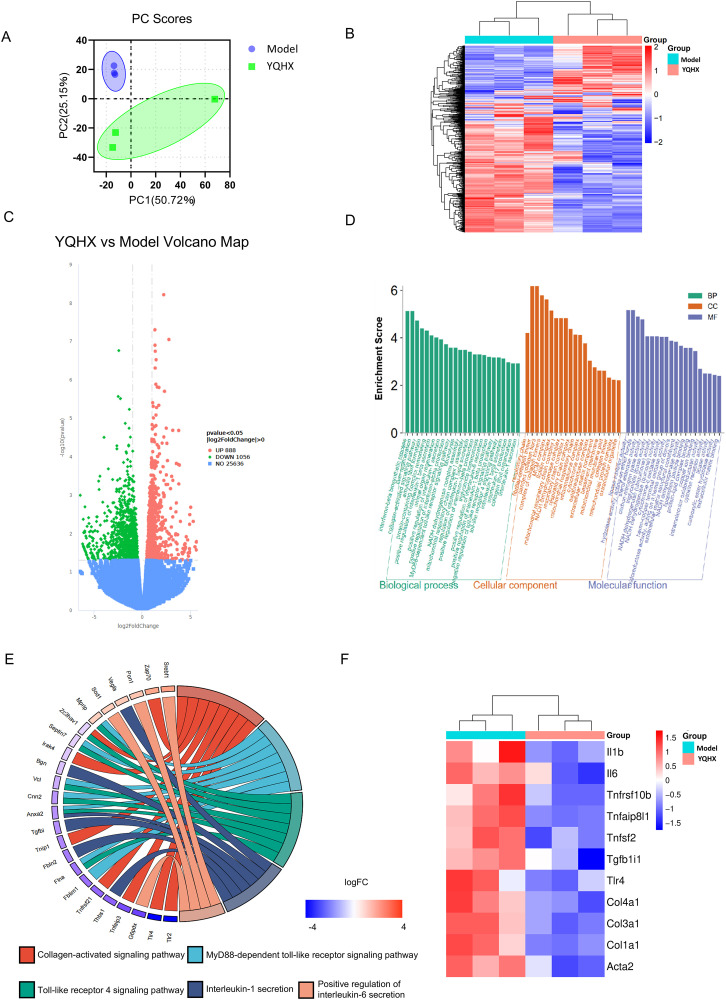
YQHX regulates several pathways related to the occurrence and development of liver fibrosis. (A) PCA analysis of gene expression values (FPKM) for all samples. (B) The x-axis represents the fold change in gene expression across groups (log2FoldChange), while the y-axis indicates the significance level of gene expression differences across groups (-log10 p value). Upregulated genes are represented by red dots, and downregulated genes are represented by green dots. (C) Hierarchical clustering of gene FPKM values using mainstream methods, with row normalization (Z-score). (D) A bar chart is drawn from the most significant terms selected from the GO enrichment analysis results. (E) A chord diagram is used to further illustrate the related pathways, with genes on the left, GO terms on the right, and connecting lines in the middle indicating hierarchical relationships. (F) Changes in the gene transcription levels of inflammatory factors and collagen fibers related to the TLR4 signaling pathway in the liver tissue of diseased mice.

In the transcriptomic analysis, it was observed that there were 888 upregulated genes and 1056 downregulated genes in mice treated with YQHX (**Fig 3A**, **3B**, **3C**) (DESeq2 p value ≤ 0.05, |log2FoldChange| ≥ 0). GO enrichment analysis revealed that pathways such as the Collagen-activated signaling pathway, MyD88-dependent toll-like receptor signaling pathway, fibrinolysis, positive regulation of interleukin-6 secretion, and toll-like receptor 4 signaling pathway were all among the top 10 pathways (**Fig 3D**, 3E). After the intervention with YQHX, the transcription levels of inflammatory factors associated with the TLR4 signaling pathway in the liver tissue of diseased mice significantly increased, while the transcription of genes related to collagen fiber formation also enhanced (**Fig 3F**).DAMPs produced by hepatocyte necrosis can activate macrophages through the TLR4/MyD88/TRAF6 pathway, leading to the transformation into M1 macrophages that produce IL-1β, IL-6, and TNF-α, promoting inflammation. On the other hand, this can activate hepatic stellate cells, promoting collagen fiber generation and further progression of liver cirrhosis.

### YQHX reduces the accumulation of M1 macrophages in the liver and the activation of the TLR4/MyD88/TRAF6 pathway

The immunofluorescence analysis of liver tissue revealed a significant increase in α-SMA deposition around the central vein in the Model Group mice compared to the control Group mice (depicted in red), which was accompanied by an elevated number of iNOS-labeled M1 macrophages (depicted in green). Treatment with YQHX reduced both α-SMA deposition and the M1 macrophage population in the liver tissue of mice with liver fibrosis (**Fig 2F**). PCR analysis indicated that YQHX downregulated the expression of TLR4 mRNA in the liver tissue (**Fig 2C**), while western blot results demonstrated its ability to attenuate the activation of the TLR4/MyD88/TRAF6 pathway in the liver tissue of mice (**Fig 2E**).

### YQHX inhibits the secretion of IL-1β, IL-6, and TNF-α by M1 macrophages induced by primary myeloid cells

**Fig 4:**
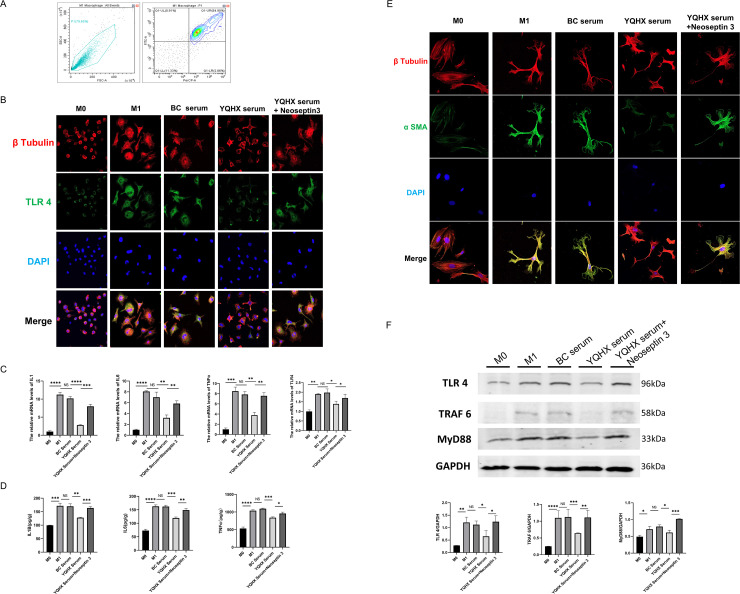
Continued.

The proportion of M1 macrophages induced by primary myeloid cells was analyzed using flow cytometry. The results indicated that the proportion of M1 cells exceeded 80% (**Fig 4A**). Immunofluorescence staining of primary macrophages treated under various conditions revealed that the induced M1 cells exhibited a larger size, adopted a crab-like morphology with pseudopods, and showed increased expression of TLR4 (depicted in green). Compared to M1 cells treated with M1 or control serum, those treated with YQHX-containing serum exhibited a significantly reduced expression of TLR4 in M1 macrophages (**Fig 4B**). These findings suggest that YQHX can inhibit the expression of TLR4 in M1 cells. Interestingly, the TLR4 agonist Neoseptin 3 reversed this inhibitory effect. PCR and ELISA were used to assess the mRNA levels of IL-1β, IL-6, and TNF-α in macrophages treated under different conditions, as well as their respective secreted contents in the supernatant. The results demonstrated that intervention with YQHX-containing serum reduced the transcriptional levels of IL-1β, IL-6, and TNF-α in M1 macrophages, with a corresponding decrease in their supernatant contents (**Fig 4C**, 4D).

### YQHX reduces the activation of HSCs indirectly co-cultured with M1 cells

To further investigate whether this phenomenon affected the activation of HSCs, we indirectly co-cultured the supernatants from macrophages treated under various conditions with primary HSCs, followed by immunofluorescence staining of the HSCs. Compared to the supernatant from M0 cells, co-culture with the supernatant from M1 cells, or M1 cells treated with the control blank serum, notable changes were induced in the cell morphology and significantly increased the expression of α-SMA in HSCs. However, co-culture with supernatant from M1 cells treated with YQHX-containing serum reduced the activation of HSCs and α-SMA expression (**Fig 4E**). Collectively, these results indicate that YQHX indirectly inhibits the activation of HSCs by suppressing the secretion of IL-1β, IL-6, and TNF-α by M1 macrophages.

### YQHX reduces the activation of the TLR4/MyD88/TRAF6 pathway in the liver tissue and M1 macrophages

Western blot analysis was performed to assess the expression levels of TLR4, MyD88, and TRAF6 in macrophages. Compared to M0 cells, M1 macrophages showed significantly increased expression of TLR4, MyD88, and TRAF6. There was no significant difference in the protein expression levels between M1 macrophages treated with the control blank serum and the untreated M1 macrophages. However, treatment of M1 macrophages with YQHX-containing serum markedly reduced the expression of TLR4, MyD88, and TRAF6 compared to those treated with the control blank serum. Moreover, a TLR4 agonist was able to reverse the inhibitory effect of YQHX on the M1 macrophages in this pathway (**Fig 4F**).

## Discussion

Because of the complexity of the multi-component and multi-target effects in TCM, network pharmacology provides a multi-layered and multidimensional approach to studying TCM, thereby enabling a deeper understanding of its pharmacological mechanisms. Initially, through network pharmacology, we predicted that the top three key proteins targeted by YQHX in alleviating liver fibrosis were IL-1β, IL-6, and TNF-α. Recent studies on the pathogenesis of liver fibrosis have indicated that the increased secretion of IL-1α/β or TNF-α by macrophages in liver fibrosis mouse models using gene knockout technology enhanced the expression of pro-fibrotic genes in HSCs, thereby exacerbating liver fibrosis [24,25]. Currently, extensive research is investigating strategies to mitigate the progression of liver fibrosis [26–28], including the use of receptor antagonists or the reduction of IL-1β and TNF-α levels in vivo [29–31]. IL-6 influences liver fibrosis progression through intricate and diverse pathways [32]. Previous studies have suggested that TCM or herbal remedies can decelerate liver fibrosis by modulating IL-6 levels in disease mouse models [33–35].

**Fig 5:**
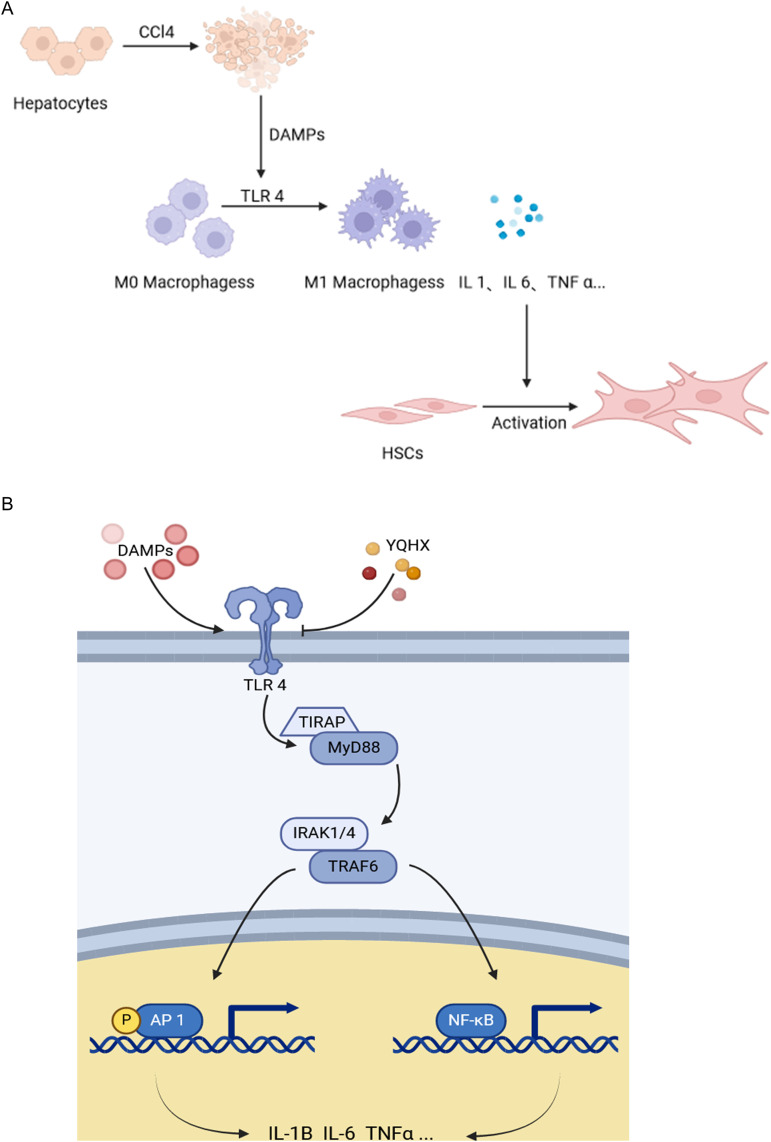
YQHX inhibits macrophage-mediated stellate cell activation in hepatitis via TLR4/TRAF6/MyD88 pathway. (A) Macrophages participate in the activation of stellate cells when hepatitis occurs. (B) YQHX can inhibit the expression of TLR4/TRAF6/MyD88 pathway and reduce the secretion of IL-1, IL-6 and TNFα by M1 macrophages.

Notably, IL-1β, IL-6, and TNF-α are inflammatory factors that are released by M1 macrophages. Factors such as viral infections, hepatic steatosis, or toxin exposure induce hepatocyte necrosis, releasing DAMPs. These DAMPs activate immune cells, such as M1 macrophages, via PRRs, thereby triggering the release of inflammatory factors. Subsequently, HSCs are activated by these factors, which leads to liver fibrosis (**Fig 5A**) [9,36]. Thus, there are grounds to believe that YQHX might alleviate local liver inflammation and fibrosis in liver fibrosis mouse models by modulating the secretion of inflammatory factors by M1 macrophages. TLR4, a classic PRR present on the surface of macrophages, serves as a transmembrane receptor pivotal for innate immune responses [37]. TLR4 recognizes exogenous ligands, such as DAMPs, on the extracellular surface of the cell membrane and concurrently interacts with corresponding ligands within the cell, initiating intricate intracellular signaling cascades. TLR4 forms complexes with TIRAP and the adaptor protein MyD88 to facilitate the interaction of IRAK1/4 with TRAF6. This interaction activates the transcription factors NF-κB and AP-1, thereby promoting the secretion of pro-inflammatory cytokines (**Fig 5B**) [38,39]. Therefore, we hypothesized that YQHX may modulate the secretion of inflammatory factors by influencing the TLR4/MyD88/TRAF6 signaling pathway in M1 macrophages. Subsequently, through molecular docking experiments and high-performance liquid chromatography, we confirmed the potential binding of these active ingredients to the therapeutic targets, as well as their ability to enter the bloodstream via oral administration in order to exert therapeutic effects.

In vivo experiments confirmed that YQHX mitigates liver inflammation and fibrosis in disease model mice and reduces the aggregation of M1 macrophages in the liver’s central vein area. YQHX decreased the expression of α-SMA and COL-1 in the liver tissues of disease model mice, as well as the expression of the key proteins MyD88 and TRAF6 in the TLR4 signaling pathway. Simultaneously, the secretion of the three inflammatory factors, IL-1β, IL-6, and TNF-α, in the liver tissue was significantly reduced. In our in vitro experiments, we selected mature macrophages derived from primary myeloid progenitor cells, which were then polarized into M1 macrophages via LPS induction. To ensure the desired proportion of M1 macrophages post-induction, we used flow cytometry analysis. Subsequently, the supernatant was collected from the macrophages subjected to various treatments and was indirectly co-cultured with primary HSCs to explore the effects of macrophages on HSCs.

The findings from our study demonstrate that YQHX inhibits the TLR4/MyD88/TRAF6 signaling pathway in M1 macrophages, which leads to reduced levels of IL-1β, IL-6, and TNF-α in the supernatant. Following co-culturing with HSCs, a decrease in the activation level of HSCs was observed. Interestingly, TLR4 agonists reversed this inhibitory effect, which suggests that YQHX achieves these effects by suppressing TLR4 activity. It is necessary to further clarify that the active ingredients of interest to us have been proven in previous studies to inhibit the progression of related liver diseases [40–42] (S2 Table 2). This primarily includes the inhibition of local inflammation in the liver [43–45] and the further activation of HSCs during inflammation [46–49], and they may even delay the progression from hepatitis to liver cancer [43,45,50]. However, this study did not conduct in vivo experiments on the screened active ingredients to confirm which one plays a major role, nor did it further investigate the specific mechanism of TLR4 inhibition. This will be a focus of our future research.

## Conclusions

In summary, this study utilized network pharmacology to predict the potential targets of YQHX for alleviating liver fibrosis. Molecular docking experiments confirmed the binding affinity of the active ingredients with the key proteins IL-1β, IL-6, TNF-α, and their upstream receptor, TLR4. High-performance liquid chromatography validated the presence of these active ingredients in YQHX. In vivo and in vitro experiments further demonstrated that YQHX suppresses the secretion of IL-1β, IL-6, and TNF-α by affecting the TLR4/MyD88/TRAF6 signaling pathway, thereby reducing HSC activation and exerting anti-fibrotic effects. This research provides new insights into the mechanisms of TCM in treating liver fibrosis, highlighting its potential use for the treatment of liver cirrhosis.

## Supporting information

S1 Text The essential parameters of the UPLC-MS Methodology. (PDF)

S2 Table Mass spectrometry data of potential effective components detected by UPLC in drug-containing serum. (PDF)

S2 Fig TIC scan pattern in positive ion mode and negative ion mode. (PDF)

S3 Table Therapeutic effects of potential active ingredients on liver diseases. (PDF)

S4 Fig Original Western Blot analysis of the target proteins expression in vivo and vitro. (PDF)

## List of abbreviations:

AST: Aspartate Aminotransferase
ALT: Alanine Transaminase
TNF-α: Tumor Necrosis Factor Alpha
α-SMA: Alpha Smooth Muscle
DAMPs: Damage Associated Molecular Patterns
PRRs: Pattern Recognition Receptors
HSCs: Hepatic Stellate Cells
TCM: Traditional Chinese Medicine
GO: Gene Ontology
KEGG: Kyoto Encyclopedia of Genes and Genomes
LPS: Lipopolysaccharide
ALP: Alkaline Phosphatase
TBIL: Total Bilirubin
GAPDH: Glyceraldehyde-3-phosphate Dehydrogenase
SDS-PAGE: Sodium Dodecyl Sulfate Polyacrylamide Gel Electrophoresis
PVDF: Polyvinylidene Fluoride
IL-1β: Interleukin 1, beta
IL-6: Interleukin 6
TNF-α: Tumor Necrosis Factor Alpha
