# Supplementary material for: Combining transcriptomics with network pharmacology to explore the mechanism of Yiqi Huoxue decoction against liver fibrosis
Source: PLoS One. 2025 Nov 26;20(11):e0337061. doi: 10.1371/journal.pone.0337061 (PMC12654879; doi:10.1371/journal.pone.0337061)
Supplement: S1 Text — (PDF) [file pone.0337061.s001.pdf]

S1 Text 1. The Essential Parameters of the UPLC-MS Methodology

The data acquisition system primarily includes Ultra Performance Liquid Chromatography (UPLC) (ExionLC™ AD, https://sciex.com.cn/) and Tandem Mass Spectrometry (MS/MS).

The liquid chromatography conditions are as follows: (1) Chromatographic column: Agilent SB-C18 1.8 μm, 2.1 mm × 100 mm; (2) Mobile phase: Phase A is ultrapure water with 0.1% formic acid, and phase B is acetonitrile with 0.1% formic acid; (3) Elution gradient: 0.00 min, B phase ratio is 5%; from 0.00 to 9.00 min, the B phase ratio increases linearly to 95%, which is maintained for 1 min; from 10.00 to 11.10 min, the B phase ratio is reduced to 5%, and equilibrated to 5% until 14 min; (4) Flow rate is 0.35 mL/min; column temperature is 40°C; injection volume is 2 μL.

The mass spectrometry conditions include: Electrospray ionization (ESI) source temperature is 500°C; ion spray voltage (IS) is 5500 V (positive ion mode) / -4500 V (negative ion mode); Ion source gas I (GSI), gas II (GSII), and curtain gas (CUR) are set at 50, 60, and 25 psi, respectively; collision-induced dissociation (CID) parameters are set to high. Triple quadrupole (QQQ) scanning uses MRM mode, and the collision gas (nitrogen) is set to medium. The declustering potential (DP) and collision energy (CE) for each MRM transition were optimized through further adjustments, and specific MRM ion pairs were monitored during each time period based on the metabolites eluted during that period.
