# Supplementary material for: Combining transcriptomics with network pharmacology to explore the mechanism of Yiqi Huoxue decoction against liver fibrosis
Source: PLoS One. 2025 Nov 26;20(11):e0337061. doi: 10.1371/journal.pone.0337061 (PMC12654879; doi:10.1371/journal.pone.0337061)
Supplement: S2 Table — (PDF) [file pone.0337061.s002.pdf]

S2 Table. Mass spectrometry data of potential effective components detected by UPLC in drug-containing serum

Components	Molecular formula	Molecular weight	Q1 (Da)	Q3 (Da)	Retention time （min）	Ionization model	Quality deviation （ppm）
Luteolin	$C_{15}H_{10}O_6$	286.24	287.06	241.05	4.5	$[M+H]^+$	-6.773962836
Hederagenin	$C_{30}H_{48}O_4$	472.7	471.35	471.35	9.1	$[M-H]^-$	0.034793827
Tanshinone IIA	$C_{19}H_{18}O_3$	294.3	295.13	277.12	9.9	$[M+H]^+$	1.047324885
Formononetin	$C_{16}H_{12}O_4$	268.26	269.08	213.09	6.2	$[M+H]^+$	0.054630424

*Q1 (Da): The molecular weight of the parent ion after the substance is added with ions by an electrospray ion source；
Q3 (Da): Characteristic fragment ions.
