# Supplementary figures and images for: Combining transcriptomics with network pharmacology to explore the mechanism of Yiqi Huoxue decoction against liver fibrosis
Source: PLoS One. 2025 Nov 26;20(11):e0337061. doi: 10.1371/journal.pone.0337061 (PMC12654879; doi:10.1371/journal.pone.0337061)

**S2 Fig 1. TIC scan pattern in positive ion mode**

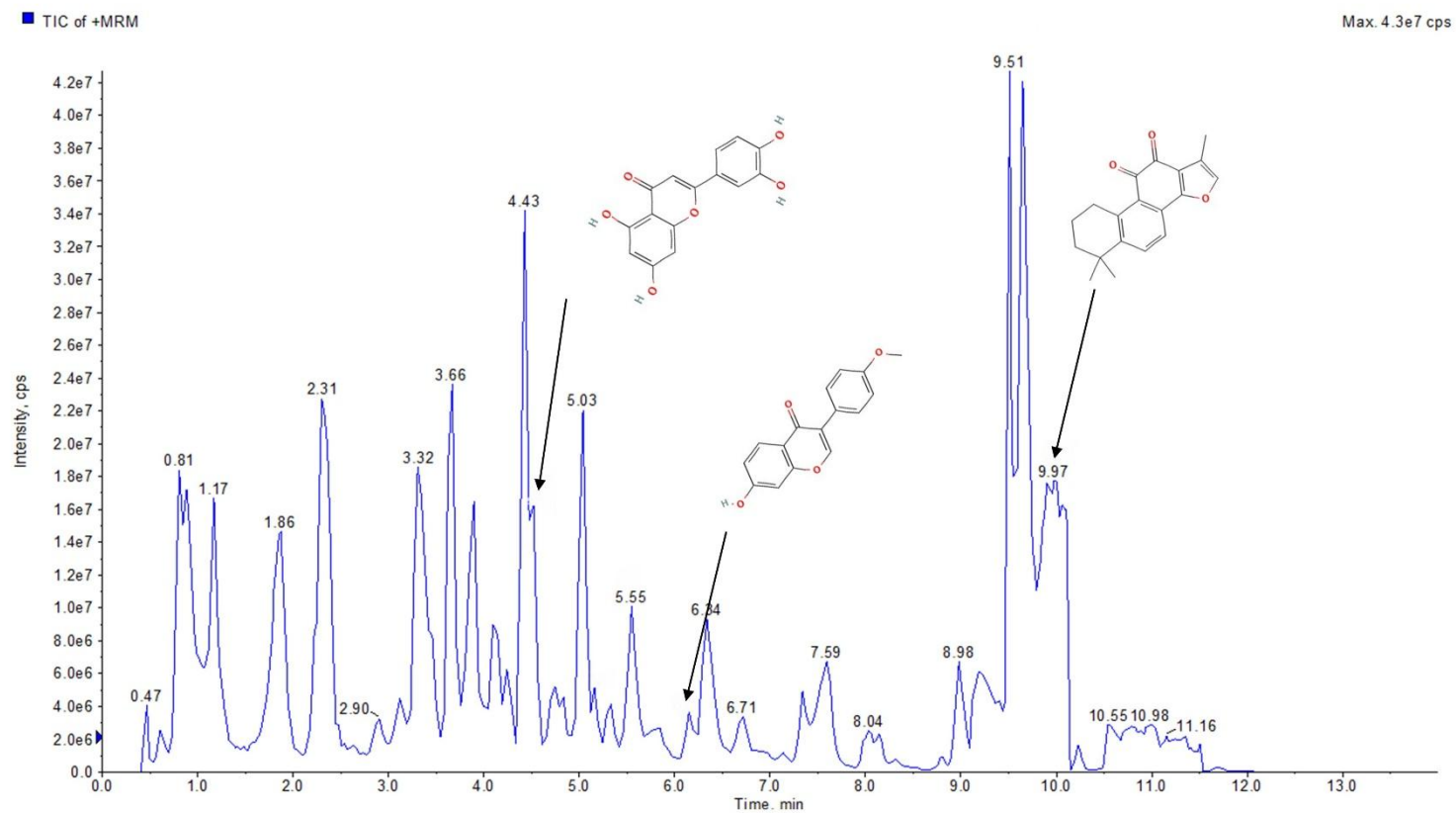

**S2 Fig 2. TIC scan pattern in negative ion mode**

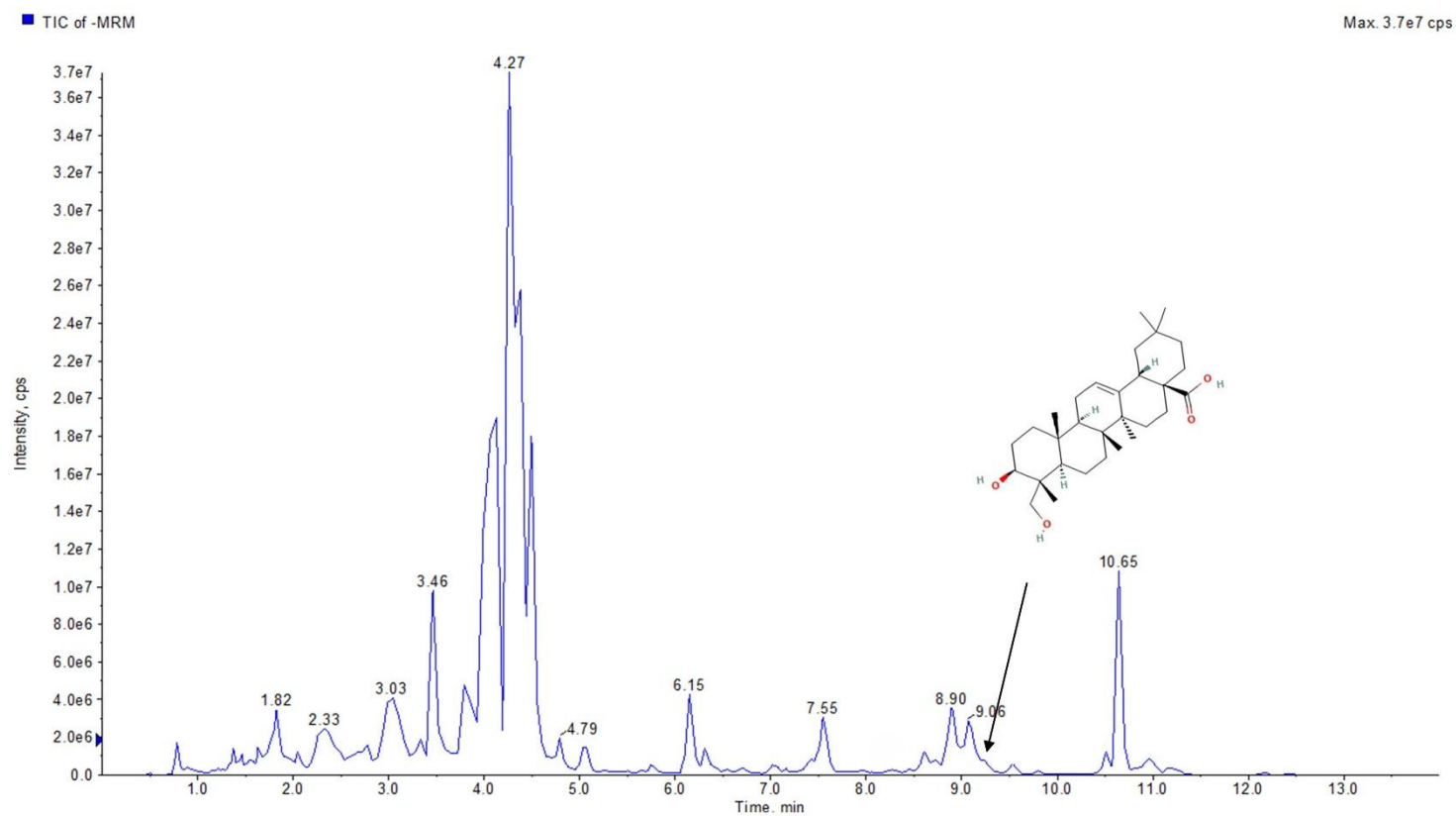

Supplement: S2 Fig — (PDF) [file pone.0337061.s003.pdf]

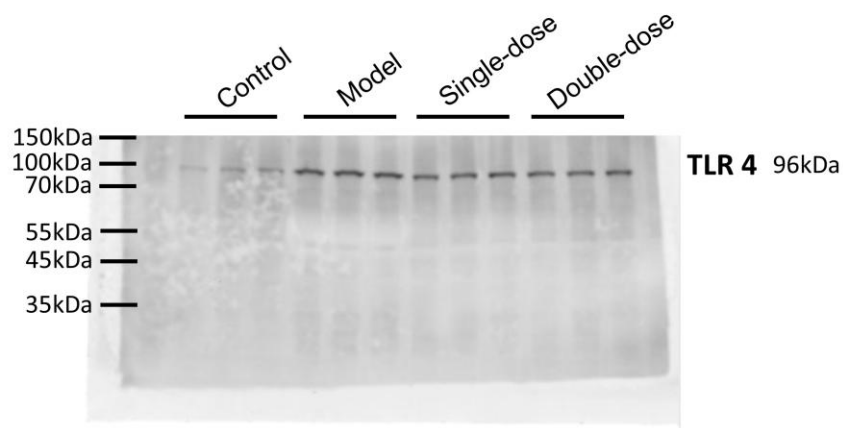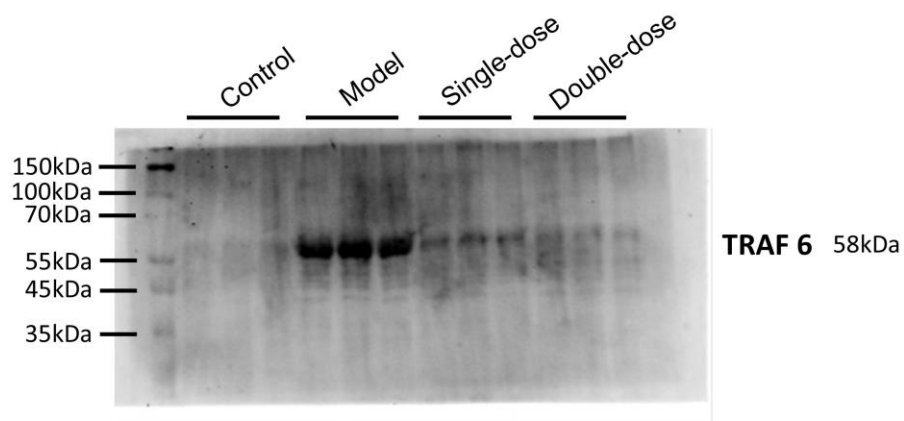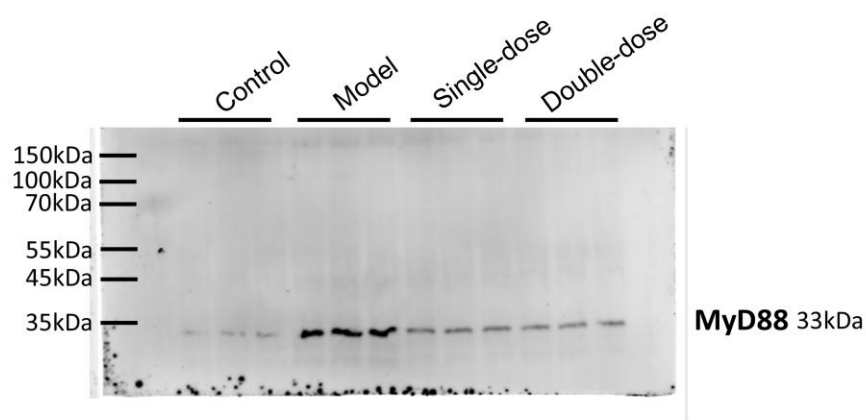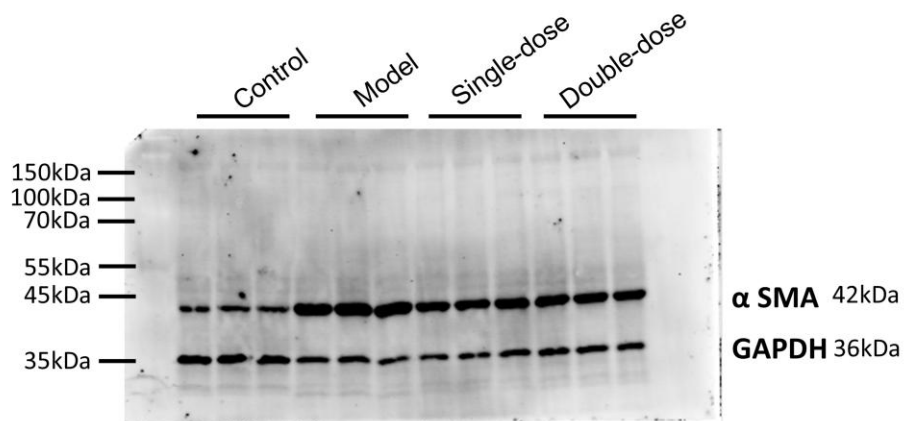

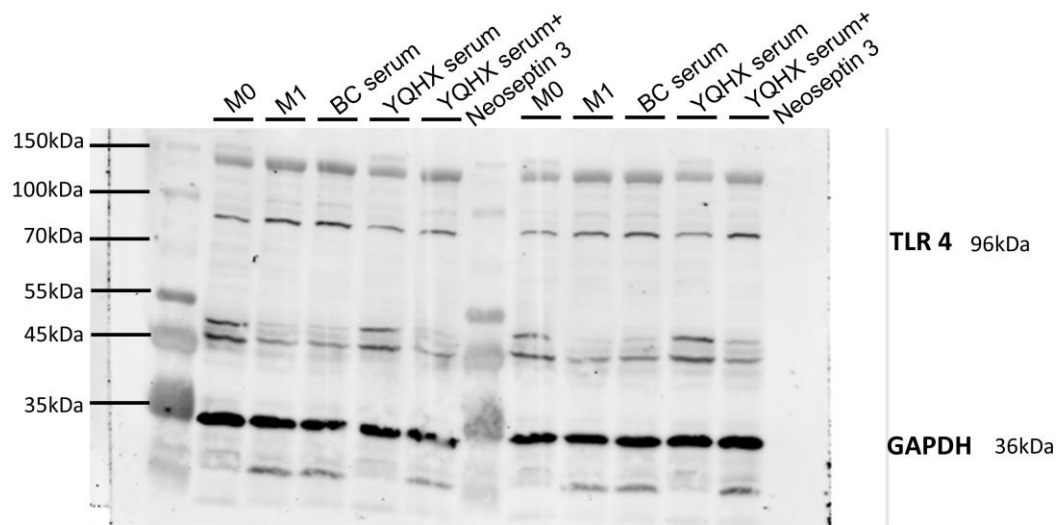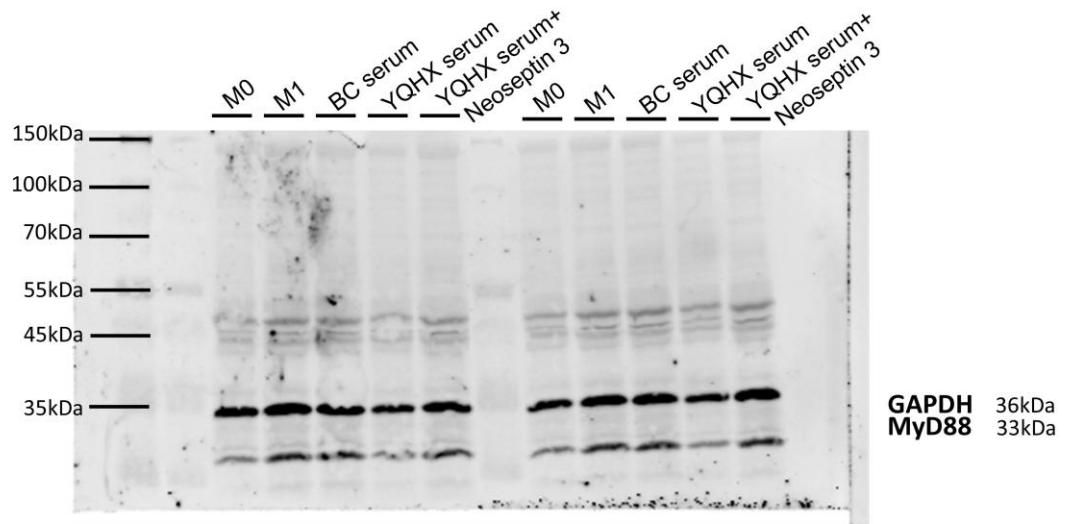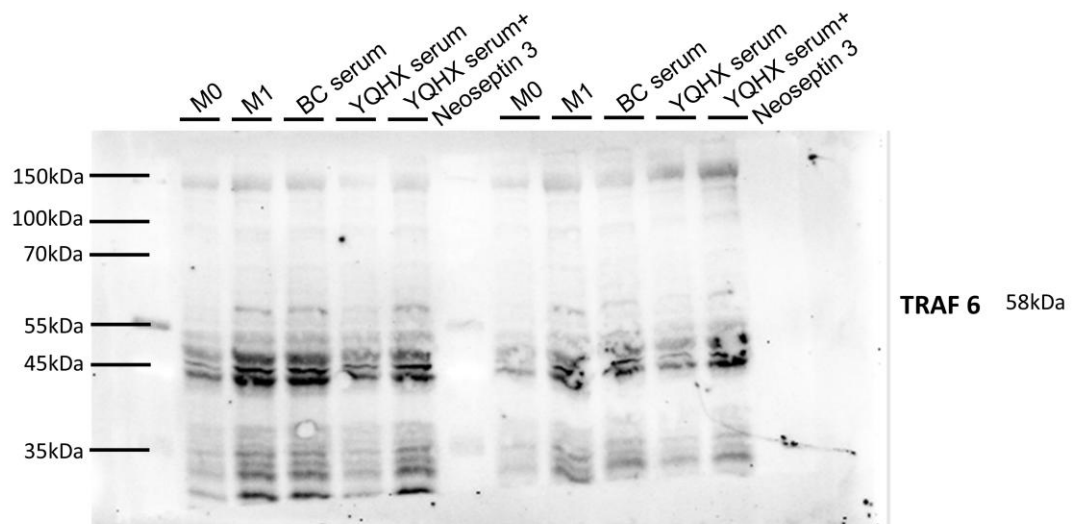

Supplement: S4 Fig — (PDF) [file pone.0337061.s005.pdf]
