# Supplementary material for: Combining transcriptomics with network pharmacology to explore the mechanism of Yiqi Huoxue decoction against liver fibrosis
Source: PLoS One. 2025 Nov 26;20(11):e0337061. doi: 10.1371/journal.pone.0337061 (PMC12654879; doi:10.1371/journal.pone.0337061)
Supplement: S3 Table — (PDF) [file pone.0337061.s004.pdf]

S3 Table. Therapeutic effects of potential active ingredients on liver diseases

Components	Molecular structural formula	Pharmacological activities	Herb sources	References
Luteolin	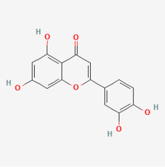	Inhibiting the activation of inflammatory corpuscles of NLRP3.<br>Detoxify.	*Radix Salviae*<br>*Alpinia Katsumadai Hayat*	(Wang et al., 2021)<br>(Wang et al., 2024)
Hederagenin	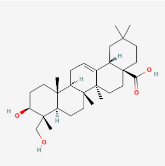	Antitumor activity.<br>Anti-inflammatory and anti-apoptosis activities.	*Poria*<br>*Hedysarum Multijugum Maxim*	(Xie et al., 2023)<br>(Zhang et al., 2024)<br>(Kim et al., 2017)
Tanshinone iia	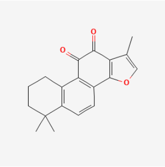	Improve the accuracy of targeting solid tumors.<br>Activating Nrf2 pathway protects liver from APAP-induced liver injury.	*Radix Salviae*	(Guo et al., 2024)<br>(Wang et al., 2016)
Formononetin	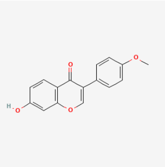	Potential active ingredients for preventing or treating liver injury.<br>Promoting HSC aging to reduce liver fibrosis.<br>Potential targets of tumor intervention.	*Hedysarum Multijugum Maxim*	(Liao et al., 2021) (Yuan et al., 2024)<br>(Liao et al., 2024) (Meng et al., 2019)
